# Modulatory Effects of Co-Fermented Pu-erh Tea with Aqueous Corn Silk Extract on Gut Microbes and Fecal Metabolites in Mice Fed High-Fat Diet

**DOI:** 10.3390/nu15163642

**Published:** 2023-08-19

**Authors:** Lin Ding, Hong Guan, Wenqing Yang, Hao Guo, Chuangang Zang, Yuchao Liu, Shan Ren, Jicheng Liu

**Affiliations:** 1 Research Center of Microecological Engineering Technology, Office of Academic Research, Qiqihar Medical University, Qiqihar 161006, China; 001229@qmu.edu.cn (L.D.); guanhong@qmu.edu.cn (H.G.); 000903@qmu.edu.cn (W.Y.); 001016@qmu.edu.cn (H.G.); 000950@qmu.edu.cn (C.Z.); 000974@qmu.edu.cn (Y.L.); 2 Basic Medical Science College, Qiqihar Medical University, Qiqihar 161006, China; renshan@qmu.edu.cn; 3 Heilongjiang Provincial Key Laboratory of Natural Medicines for Anticancer, Qiqihar Medical University, Qiqihar 161006, China

**Keywords:** co-fermented Pu-erh tea, corn silk, gut microbiota, fecal metabolites, high-fat diet

## Abstract

Pu-erh tea is recognized for its weight loss effects, but its potential association with gut microbiota and metabolites remains unclear. This research explored the alterations in gut flora and metabolite composition upon treatment with a co-fermented Pu-erh tea with an aqueous corn silk extract (CPC) in obese mice by employing integrated 16S ribosomal RNA gene sequencing and untargeted metabolomics processes. For 8 weeks, mice were fed control, high-fat, and high-fat diets which included a 46 mg/mL CPC extract. The CPC extract the alleviated high-fat diet (HFD), it stimulated systemic chronic inflammation, and it reduced the body weight, daily energy consumption, and adipose tissue weight of the mice. It also modified the gut microbiota composition and modulated the *Lactobacillus*, *Bifidobacterium*, *Allobaculum*, *Turicibacter*, and *Rikenella genera*. Fecal metabolomics analysis revealed that the CPC extract influenced the caffeine, cysteine, methionine, tryptophan, biotin metabolism pathways, primary bile acid, and steroid biosynthesis. This research revealed that the CPC extract could inhibit HFD-stimulated abnormal weight gain and adipose tissue accumulation in mice, and modulate mice gut microbiota composition and multiple metabolic pathways.

## 1. Introduction

Obesity is a global epidemic that endangers human health. It is a metabolic disorder induced by many genetic and non-genetic factors. Obesity enhances the occurrence and progression of several diseases, including cardiovascular disease (CVDs), hypertension, and diabetes [1]. The chronic intake of high-fat diet (HFD) can lead to obesity and weight gain, given the accumulation of abnormal lipids in the liver and low levels of systemic inflammation [2]. HFDs play a crucial role in the development of metabolic syndrome-related diseases by altering the gut microbiota (GM). The fecal microbiome and its metabolites are believed to affect the host’s lipid metabolism [3]. Some bacteria and their metabolites, such as bile acids (BAs) or short-chain fatty acids (SCFAs), can modify energy harvesting efficiency and contribute to increased vulnerability to metabolic diseases [4,5].

Pu-erh tea, a traditional Chinese tea exclusively produced in the Yunnan Province, has a long history [6]. It is made from sun-dried Yunnan large-leaf teas that undergo microbial pile-fermentation. Pu-erh tea offers various health benefits, including the alleviation or reversal of hypercholesterolemia, hyperlipidemia, and obesity. The consumption of Pu-erh tea has been found to significantly reduce body weight and fat weight, with documented anti-obesity effects in rats, mice, and humans. However, the underlying mechanisms of these effects have yet to be fully identified. Proper fermentation is the basis of developing quality Pu-erh tea. In essence, the chemical substances in the tea leaves undergo a series of complex chemical reactions such as transformation, isomerization, degradation, polymerization, and coupling under the combined effects of enzymatic promotion, moisture, and heat; this is because of the effects of microbial growth and metabolism, which contributes to the unique quality of Pu-erh tea [7]. Currently, there is significant interest in exploring new fermentation processes for Pu-erh tea, such as Ganpu tea, which involves co-fermentation with Citrus peel and is widely popular in the Chinese market [8].

Thus, the objective of this research was to determine the ameliorative effects of co-fermented Pu-erh tea with aqueous corn silk extract (CPC) on HFD-triggered obesity in mice, and to understand its effects on the microbial community and metabolite composition in feces.

## 2. Materials and Methods

### 2.1. Materials and Reagents

The control diet was composed of 10% lipids, 20% protein, and 70% carbohydrates, whereas the HFD contained 40.29%, 17.71%, and 42% of these nutrients, respectively (Beijing HFK Bioscience Co., Ltd., Beijing, China). The composition of the HFD and control diet is displayed in Table 1. The mouse insulin (INS), tumor necrosis factor-α (TNF-α), and interleukin-6 (IL-6) ELISA Kit were purchased from Shanghai Jianglai Biotechnology (Shanghai, China).

### 2.2. Preparation of CPC Extract

To prepare the corn silk extract, a vacuum-reflux extraction and concentration unit (Chengdong Medicine Machine Instrument Factory, Wenzhou, China) were used. Approximately 20 kg of dried corn silk was extracted three times with boiling water (1:15 *w*/*v*) using this unit. Each extraction lasted for at least 60 min. Subsequently, the concentrated solution was spray dried (GEA Spray Drying Equipment, Dusseldorf, Germany) to obtain the corn silk extract. For the preparation of the CPC, Sun-dried Yunnan large-leaf teas were mixed with corn silk extract in a 5.25:100 (*w*/*w*) ratio. The mixture was then placed in a stainless-steel fermentation container. Eight layers of wet gauze were used to cover the mixture, and the container was further covered with a lid. The fermentation container was placed in a fermentation chamber at a temperature of 55 ± 2 °C, and a relative humidity of 32 ± 2% was used for a fermentation period of 23 days. Throughout this period, the fermentation mixture was turned 3 to 5 times, and the water content of the fermentation mixture was controlled to 36 ± 2% (*w*/*w*) using proper rehydration methods.

After the completion of fermentation, the fermentation mixture was ventilated, dried, and then stored. The CPC extract was prepared by subjecting approximately 6 kg of dried CPC to vacuum reflux extraction, using the vacuum reflux extraction and concentration unit containing 120 L boiling water for 90 min each time; this process was repeated three times. The extract powder was obtained using spray-drying. The CPC extract powder yield was 19.5%. The polysaccharide contents in the extract were determined using the phenol–sulphuric acid method; the free amino acid content was determined using the ninhydrin coloring method; the total catechin, epigallocatechin gallate (EGCG), epicatechin gallate (ECG), gallic acid, and caffeine content was estimated by employing high-performance liquid chromatography; the total phenolic content was measured with the help of the folin-ciocalteu method (Standardization Administration of the People’s Republic of China. GB/T 8313-2018). The CPC extract contained 5.81% (*w*/*w*) polysaccharide, 1.87% (*w*/*w*) free amino acid, 1.40% (*w*/*w*) total catechin, 0.22% (*w*/*w*) EGCG, 0.73% (*w*/*w*) ECG, 0.48% (*w*/*w*) gallic acid, 3.58% (*w*/*w*) caffeine, as well as 10.46% (*w*/*w*) total phenolic content.

### 2.3. Animals and Experimental Design

Animal experimentation was assessed and authorized by the Qiqihar Medical University’s Animal Ethical Care Committee (approval number: QMU-AECC-2020-69). All animal-based experimental procedures were carried out at the Laboratory Animal Centre of Qiqihar Medical University, China, following national regulations and local guidelines. Male C57BL-6J mice, which weighed 18 ± 2 g, were purchased from Changsheng Experimental Animal Co., Ltd. (Changchun, China). Under controlled conditions, the mice were kept in a SPF habitat with a 12 h light/dark cycle at 20 °C to 22 °C, with 45 ± 5% humidity. The mice were fed a control diet for a week, followed by random division into three groups (*n* = 8). The mice in the HFD-fed group were administered either a HFD with ultrapure water (10 mL/kg) or an aqueous solution of CPC extract (46 mg/mL), whereas mice in the control (Ctrl) group received a standard diet with ultrapure water (10 mL/kg). The CPC extract was administered daily at 460 mg/kg. The mice had unlimited food and water for eight weeks, and their body weight and food consumption were recorded every seven days. Following one night fast, all mice were euthanized at the end of the experiment, and blood and fecal samples were obtained for subsequent analytical testing. The wet weights of the mice liver and epididymal tissues were recorded, and some tissues were subjected to fixation with 4% paraformaldehyde, whereas the rest were placed in liquid nitrogen.

### 2.4. Biochemical Indicator Measurement

Total cholesterol (TC) level, triglyceride (TG) level, low-density lipoprotein cholesterol (LDL-C) levels, high-density lipoprotein cholesterol (HDL-C) levels, alanine aminotransferase (ALT) levels, and aspartate aminotransferase (AST) levels were measured with the help of an auto chemical analyzer (Olympus AU 600, Tokyo, Japan). ELISA kits were used to measure the levels of INS, IL-6, and TNF-α in the serum and liver.

### 2.5. Histopathological Examination

The epididymal tissue samples were removed from the 4% paraformaldehyde solution after 24 h and embedded in paraffin; then, they were sectioned and stained using hematoxylin and eosin. Changes in the histological images were observed under a light microscope (Olympus CX31, Tokyo, Japan).

### 2.6. Gut Microbiota Analysis

Following the initial isolation of the Microbial DNA from the fecal samples, the V3-V4 region of the bacterial 16S ribosomal RNA (16S rRNA) gene was subjected to amplification using a primer PCR, followed by sequencing via the MiSeq platform (Illumina, Inc., San Diego, CA, USA). The specific experimental methods and analyses are given in prior research [9].

### 2.7. Untargeted Fecal Metabolomic Analysis

The untargeted fecal metabolomic analysis protocols are explained in prior research [9]. The general process is as follows. Liquid chromatography with tandem mass spectrometry analysis adopts the UPLC system containing the UPLC BEH Amide column and Q Active HFX mass spectrometry (Orbitrap MS, Thermo Fisher Scientific, Waltham, MA, USA). Using information-dependent acquisition, the mass spectra were obtained using a QE HFX mass spectrometer under the control of the acquisition software (Xcalibur 4.0). ProteoWizard 3.1.100 was employed for the conversion of the original data into the mzXML format, and the metabolites were then annotated using the internal MS2 database (Biotree DB V2.1).

### 2.8. Data Analysis

Data are shown as mean ± standard deviation. Differences between multiple groups were assessed using one-way ANOVA and IBM SPSS Statistics version 26. Levene’s test was used to verify variance homogeneity. LSD (assuming equal variance), or Tamhane’s T2 (not assuming equal variance), post hoc tests were used to determine differences between groups (*p* < 0.05). The correlations found between variables were identified via Pearson’s product–moment correlation coefficient. Differences between the individual indices of α-Diversity were found using the Krushal–Wallis test. Differences in species diversity between groups were analyzed in conjunction with PCoA and Adonis test. GraphPad Prism version 8.0 was employed to create the graphs. *p* < 0.05 indicated the significance level.

Multivariate analysis was performed using SIMCA 16.0.2. Principal Component Analysis (PCA) was used to show the distribution and grouping of the samples. Orthogonal projections to latent structures–discriminate analysis (OPLS-DA) was applied to find significantly changed metabolites. Furthermore, the value of variable importance in the projection (VIP) of the first principal component in OPLS-DA was obtained. The metabolites with VIP > 1 and *p* < 0.05 (Student’s *t*-test) were considered as significantly changed metabolites.

## 3. Results

### 3.1. Impact of the CPC Extract on Body Weight, Food Intake, and Energy Intake

**Figure 1:**
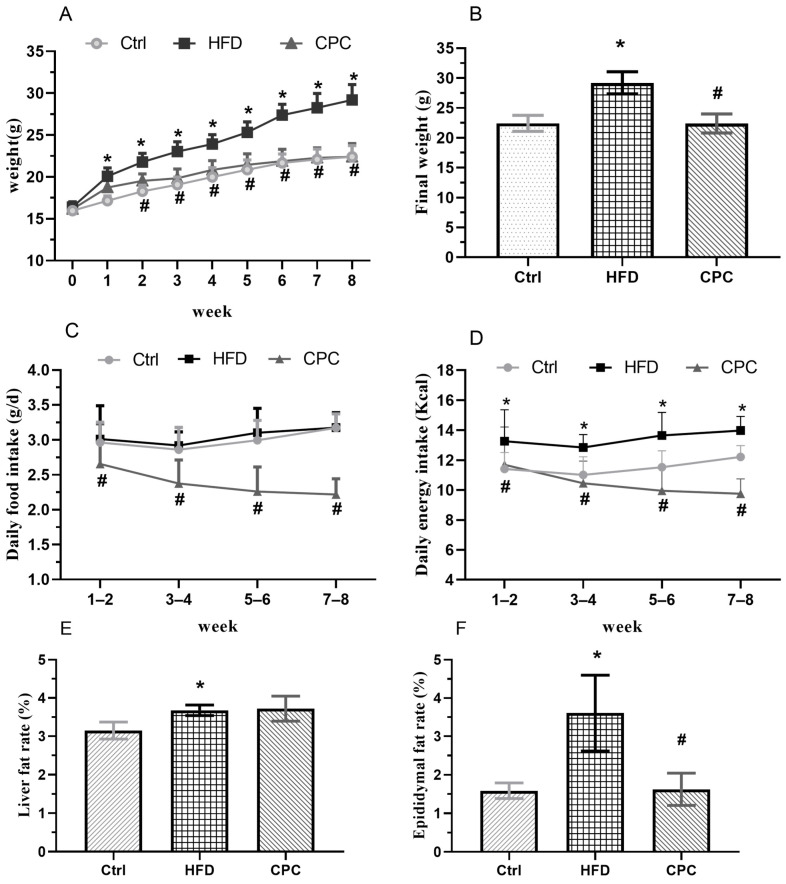
Impact of the CPC extract on body weight, energy intake, liver weight index (g/100 g body weight), and epididymal fat weight index (g/100 g body weight). (**A**) Body weight; (**B**) final weight; (**C**) daily food consumption; (**D**) daily energy consumption; (**E**) liver rate; (**F**) epididymal fat rate. Ctrl, control. HFD, high-fat diet. CPC, co-fermented Pu-erh tea with aqueous corn silk extract. The data are presented as means ± SD (*n* = 8). * *p* < 0.05 versus Ctrl, # *p* < 0.05 versus HFD.

The 8-week dietary intervention resulted in a remarkable rise in the body weight of the HFD mice in contrast with the control group, whereas the CPC extract reversed this increase in weight. The daily food intake and energy intake were remarkably decreased among the CPC group mice compared with the HFD-fed mice (Figure 1).

### 3.2. Impact of the CPC Extract on Epididymal Fat Tissue Mass and Serum Lipid Levels

**Figure 2:**
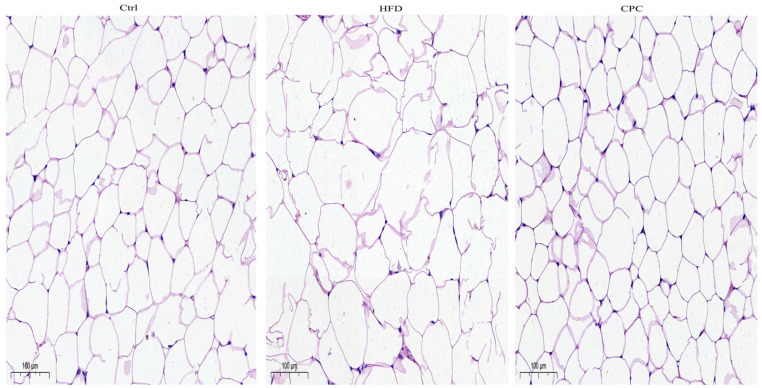
Impact of the CPC extract on the histopathology of adipose tissue in mice (200× original magnification). Ctrl, control. HFD, high-fat diet. CPC, co-fermented Pu-erh tea with aqueous corn silk extract.

The liver and epididymal fat tissue weights were remarkably enhanced in the HFD group in contrast to the control group (Figure 1F), whereas the CPC extract inhibited a rise in epididymal fat tissue weight (*p* < 0.05). Moreover, the adipose tissue cells of the HFD-fed mice were remarkably larger compared with those of the control group mice, and supplementation with the CPC extract reversed the observed tissue enlargement (Figure 2).

**Figure 3:**
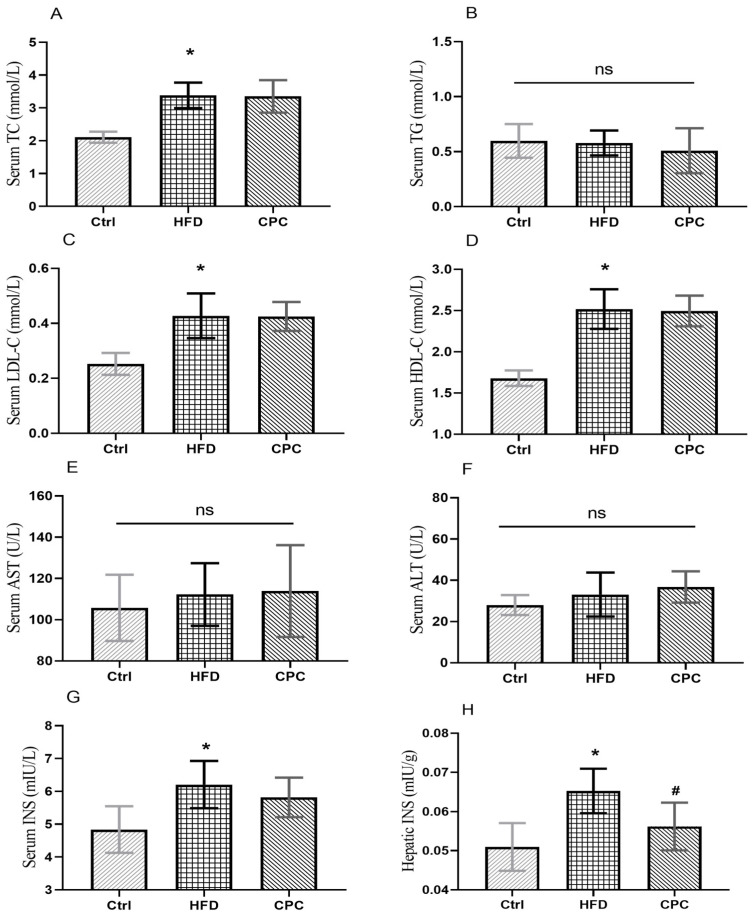
Impact of the CPC extract on serum lipid parameters, liver function indicators, and insulin levels. (**A**) Serum TC level; (**B**) serum TG level; (**C**) serum LDL-C level; (**D**) serum HDL-C level; (**E**) serum AST level; (**F**) serum ALT level; (**G**) serum INS level; (**H**) hepatic INS level. Ctrl, control. HFD, high-fat diet. CPC, co-fermented Pu-erh tea with aqueous corn silk extract. TC, total cholesterol. TG, triglyceride. LDL-C, low-density lipoprotein cholesterol. HDL-C, high-density lipoprotein cholesterol. ALT, alanine aminotransferase. AST, aspartate aminotransferase. INS, insulin. The data are presented as means ± SD (*n* = 8). * *p* < 0.05 versus Ctrl, # *p* < 0.05 versus HFD, ns no significant.

The serum levels of TC and LDL-C were significantly increased in the HFD group compared with the control group (Figure 3). The HDL-C levels were remarkably enhanced in HFD group compared with the Ctrl group (*p* < 0.05), and the CPC extract did not have an impact on this change. Serum TG, ALT, and AST levels showed no difference between groups. The serum and liver tissue insulin levels were remarkably increased in the HFD group in contrast with the control group, whereas the liver tissue insulin levels were remarkably reduced in the CPC group (*p* < 0.05).

### 3.3. Impact of the CPC Extract on Chronic Inflammation

**Figure 4:**
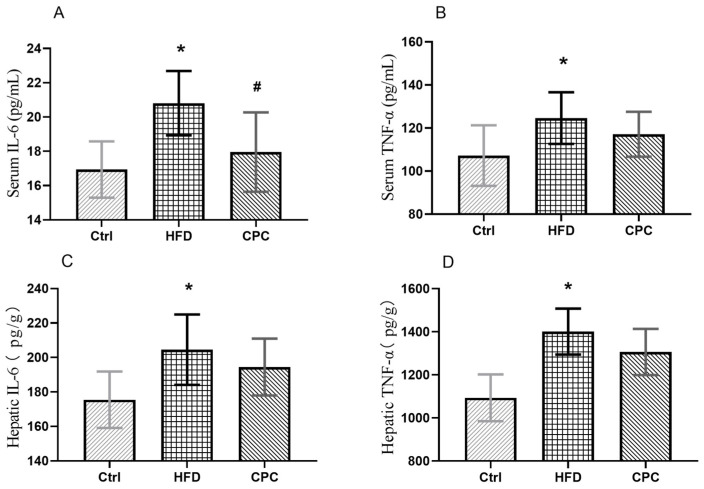
Impact of the CPC extract on inflammation. (**A**) Serum IL-6 level; (**B**) serum TNF-α level; (**C**) hepatic IL-6 level; (**D**) hepatic TNF-α level. Ctrl, control. HFD, high-fat diet. CPC, co-fermented Pu-erh tea with aqueous corn silk extract. TNF-α, tumor necrosis factor-α. IL-6, interleukin-6. The data are presented as means ± SD (*n* = 8). * *p* < 0.05 versus Ctrl, # *p* < 0.05 versus HFD.

The serum, liver IL-6, and TNF-α levels were remarkably elevated in the HFD group compared with the Ctrl group, indicating chronic inflammation in the mice (*p* < 0.05). The serum IL-6 levels were remarkably lowered in the CPC group compared with the HFD group (Figure 4).

### 3.4. Impact of the CPC Extract on Diversity and Composition of GM

**Figure 5:**
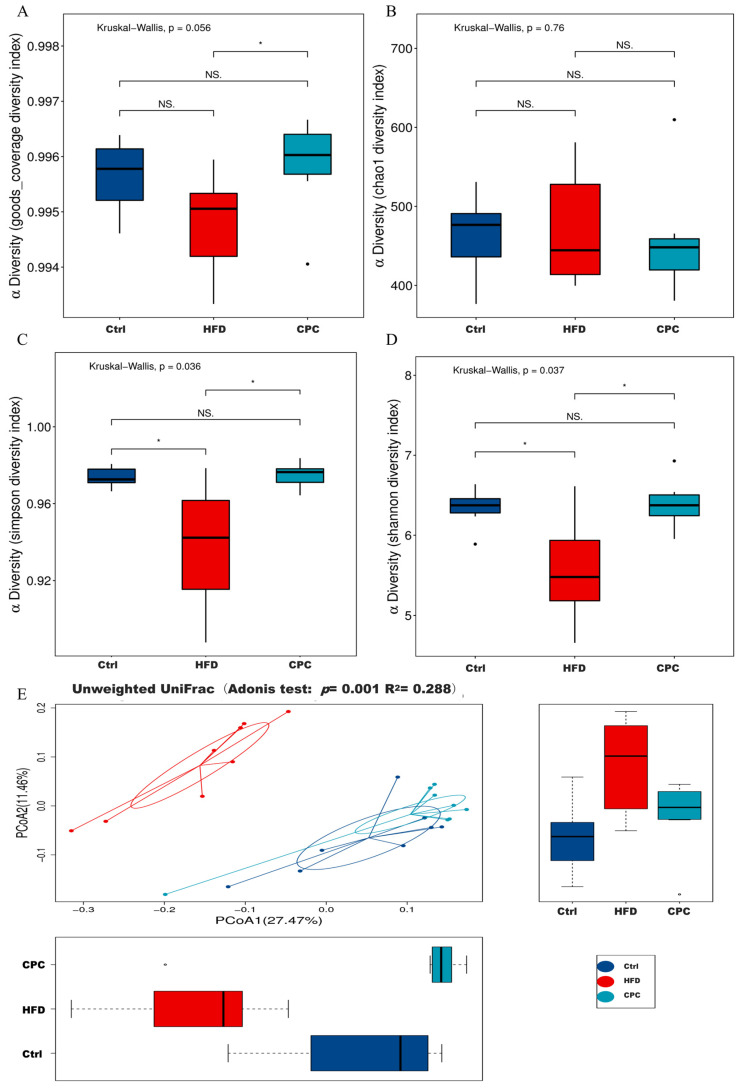
Impact of the CPC extract on the α-diversity and β- diversity indexes of the GM in mice. (**A**–**D**) α-Diversity; (**E**) unweighted UniFrac PCoA analysis. Ctrl, control. HFD, high-fat diet. CPC, co-fermented Pu-erh tea with aqueous corn silk extract. The data are presented as means ± SD (*n* = 8). * *p* < 0.05 versus the other groups, ns no significant.

The regulatory impacts of the CPC extract on the GM in mice were investigated by sequencing the bacterial 16S rRNA gene in the feces of each group (*n* = 8). A total of 858,820 clean reads with a 416 bp in average length were obtained and clustered into 1099 operational taxonomic units with a 97% similarity. Regarding the α-diversity index results, the Simpson and Shannon diversity index levels appeared remarkably lower in the HFD group compared with the control group, whereas the CPC group exhibited the opposite trend (Figure 5A,D). UniFrac PCoA analysis exhibited remarkable variations in bacterial flora structure in both the control and CPC groups, as compared with the HFD group (Figure 5E).

**Figure 6:**
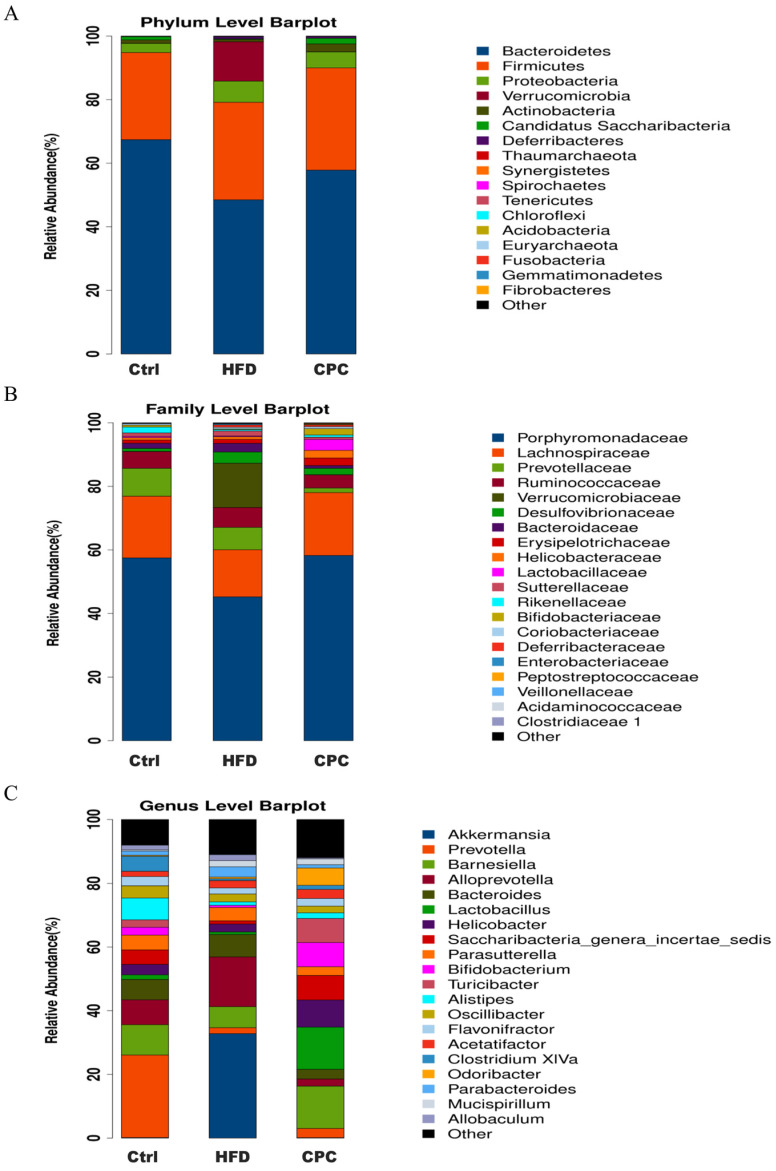
Effects of CPC on the gut microbiota composition in mice. Microbial community bar plot at (**A**) the phylum level; (**B**) the family level; (**C**) the genus level. Ctrl, control. HFD, high-fat diet. CPC, co-fermented Pu-erh tea with aqueous corn silk extract.

Bacteroidetes, Proteobacteria, Verrucomicrobia, Actinobacteria, and Firmicutes were the dominant phyla in the mouse gut flora. The abundance of Firmicutes, Verrucomicrobia, and Proteobacteria was increased, whereas that of Bacteroidetes and Actinobacteria was decreased in the HFD group, in contrast to the control group. Conversely, the abundance of Bacteroidetes and Actinobacteria improved, and that of Verrucomicrobia and Proteobacteria decreased in the CPC group, reversing the HFD-induced GM dysbiosis (Figure 6A). In contrast with the control group, the HFD group showed a remarkable improvement in terms of the abundance of Verrucomicrobiaceae, Bacteroidaceae, and Desulfovibrionaceae, but it showed a reduction in terms of the abundance of Porphyromonadaceae and Lachnospiraceae at the family level. Similarly, the CPC extract reversed the HFD-induced gut microbiota imbalance at the family level. The gut microbiota results for each group at the genus level are shown in Figure 6C.

**Figure 7:**
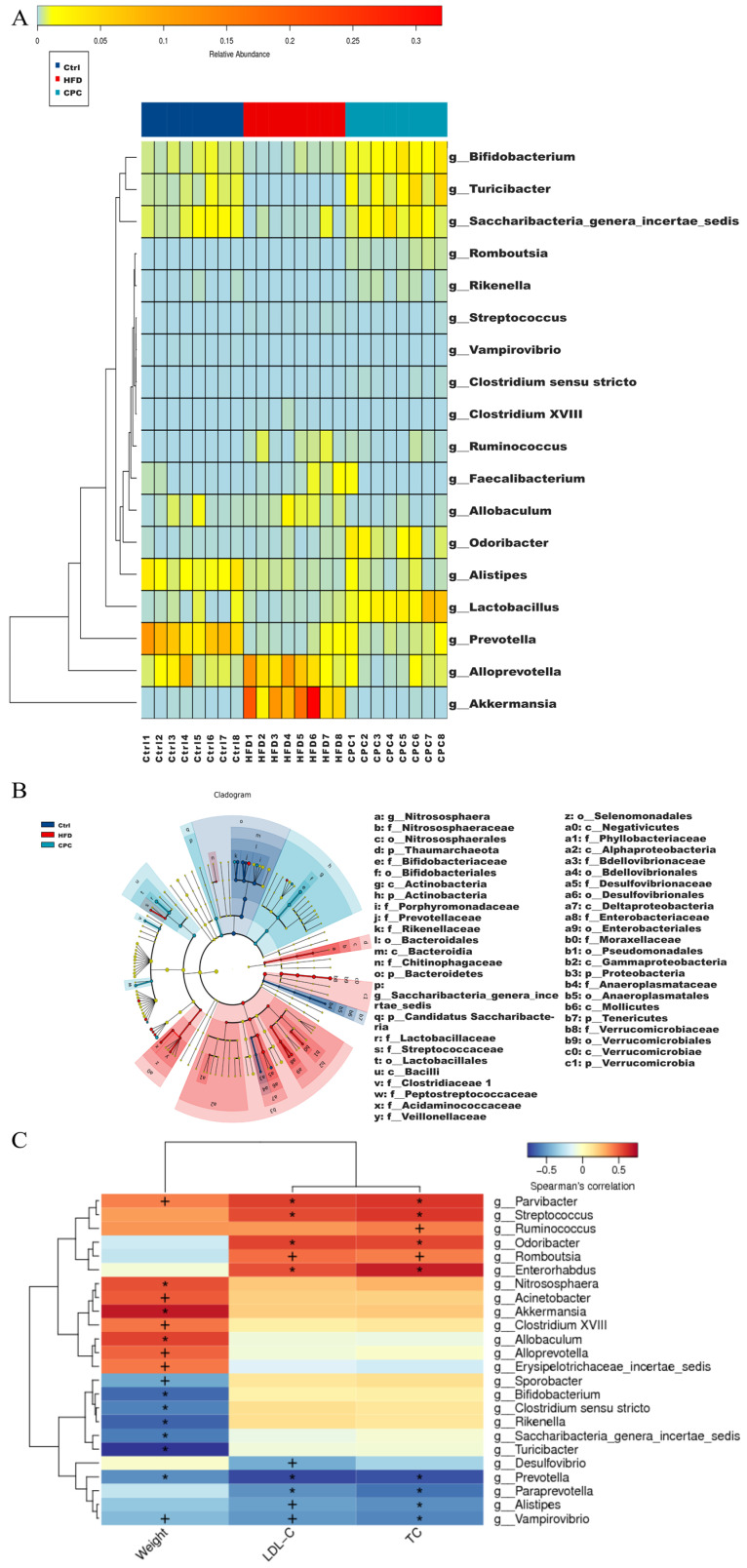
Comparative analysis of the community composition of gut microbiota. (**A**) Heatmap of remarkable variations in the microbiota at the genus level; (**B**) LDA EffectSize, different colours indicate different subgroups, and nodes of different colours indicate microbiota that play an important role in the subgroup represented by that colour, yellow nodes indicate microbiota that do not play an important role in the different subgroups.; (**C**) Spearman correlations concerning obesity symptoms and GM. Red cubes depict positive correlations, and blue cubes depict negative correlations. Ctrl, control. HFD, high-fat diet. CPC, co-fermented Pu-erh tea with aqueous corn silk extract. TC, total cholesterol. LDL-C, low-density lipoprotein cholesterol. Significance is marked by + when *p* < 0.05 and by * when *p* < 0.01.

Figure 7A shows the bacterial groups at the genus level that differed significantly between the HFD and CPC groups, including Akkermansia, Alistipes, Allobaculum, Alloprevotella, Bifidobacterium, Clostridium XVIII, Clostridium sensu stricto, Faecalibacterium, Lactobacillus, Odoribacter, Prevotella, Rikenella, Romboutsia, Ruminococcus, Saccharibacteria_genera_incertae_sedis, Streptococcus, Turicibacter, and Vampirovibrio. In the LEfSe analysis, each group was given a different colour, with the coloured nodes in that group indicating colonies that made a significant contribution to group variability, and the yellow nodes indicating colonies that had little or no effect (Figure 7B).

Next, Spearman correlation analysis was performed to evaluate the associations between the differential bacterial flora at the genus level and mouse body weight and serum TC and LDL-C levels to determine the bacteria associated with obesity symptoms (Figure 7C). The abundance of *Allobaculum*, *Alloprevotella*, *Parvibacter*, *Clostridium XVIII*, *Akkermansia*, *Acinetobacter*, *Nitrososphaera*, and *Erysipelotrichaceae_incertae_sedis* were positively correlated with body weight (*p* < 0.05). In addition, *Rikenella*, *Saccharibacteria_genera_incertae_sedis*, *Turicibacter*, *Clostridium sensu stricto*, *Bifidobacterium*, *Sporobacter*, and *Prevotella* had a remarkable negative correlation with body weight (*p* < 0.05).

### 3.5. Impact of the CPC Extract on Fecal Metabolites

In accordance with the metabolic curves shown in Figure S1, the principal component analysis score plots in the positive (POS) and negative (NEG) modes clearly distinguished between the HFD and CPC groups. The R^2^Y and Q^2^ for the HFD and CPC groups were 0.954 and 0.828 for the POS ion model, and 0.9118 and 0.707 for the NEG ion model, respectively. The R^2^Y values were both >0.5, indicating a reliable and stable model. Each point in the volcano plot represents one metabolite. Larger scatters indicate larger VIP values, remarkably up-regulated metabolites are shown in red, remarkably down-regulated metabolites are shown in blue, and non-significantly different metabolites are shown in grey in the scatter, reflecting the final results of the screening test. The tandem mass spectrometry fragmentation models were matched to the structural information of the metabolites using the Human Metabolome Database (HMDB) and the Kyoto Encyclopedia of Genes and Genomes Database (KEGG). Screening for metabolites on the database was conducted using VIP > 1 and *p* < 0.05 as the screening thresholds. A total of 583 differential metabolites were analysed between the control and HFD groups, of which, 478 and 105 were found in POS and NEG ion modes, respectively. A total of 237 differential metabolites were analyzed between the CPC and HFD groups, of which, 212 and 25 metabolites were found in POS and NEG ion modes, respectively (Figures S2 and S3). 

### 3.6. KEGG Metabolic Pathway Analysis

**Figure 8:**
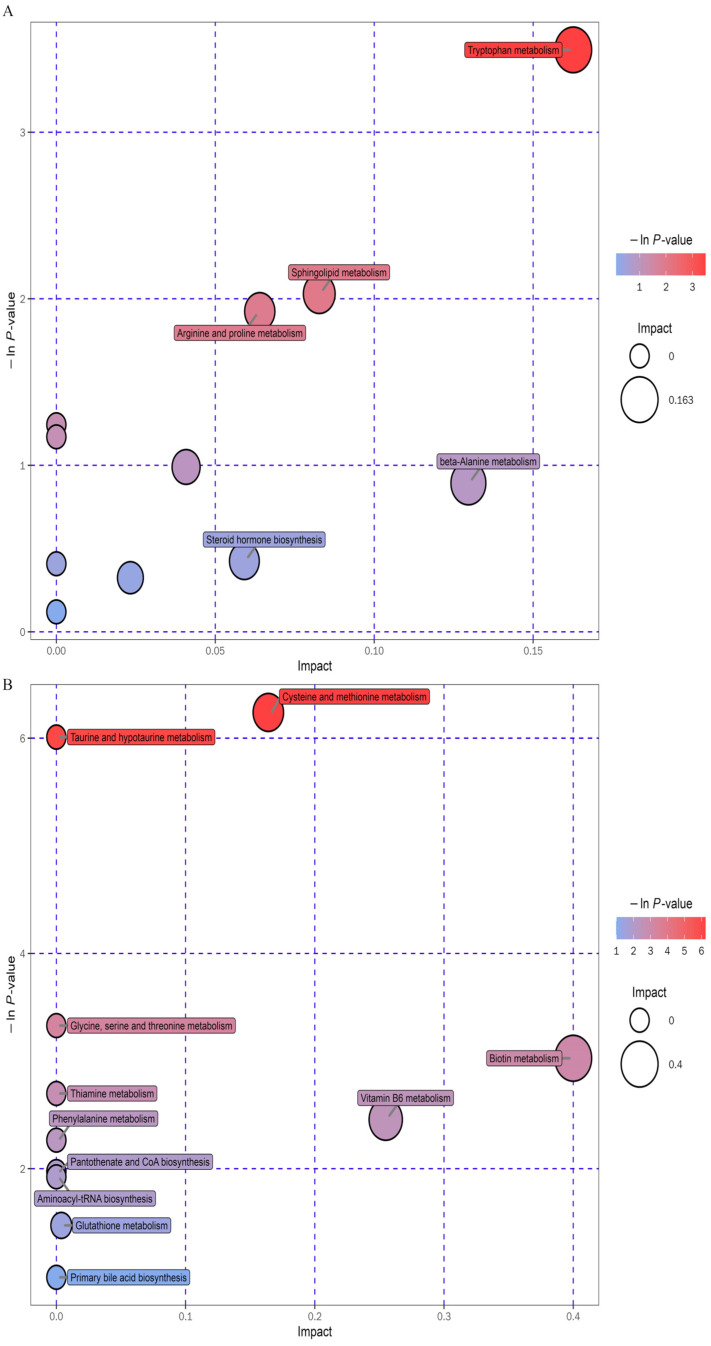
Modulation of fecal metabolic pathways in HFD mice using CPC extracts. Bubble plot map ((**A**) POS ion, (**B**) NEG ion). HFD, high-fat diet. CPC, co-fermented Pu-erh tea with aqueous corn silk extract. POS, positive. NEG, negative.

The above metabolites were identified from the Human Metabolome Database and grouped into 11 subclasses, including the following: neolignans and related compounds; benzenoids; nucleosides, nucleotides, and analogs; lignans, alkaloids, and derivatives; lipids and lipid-like molecules; hydrocarbon derivatives; organoheterocyclic and organic nitrogen compounds; organic acids and derivatives; organooxygen compounds and phenylpropanoids and polyketides. The results of the pathway study are presented in the form of bubble plots. The *p*-value of the enrichment analysis is indicated by the vertical coordinate and the colour of the bubble, where the deeper the colour, the lower the *p*-value, and the greater the significance of the enrichment (Figure 8A,B). 

Regulation of a total of 11 potential pathways was observed after treatment with the CPC extract in the POS ion mode, as follows: tryptophan and sphingolipid metabolism; steroid hormone biosynthesis phenylalanine and caffeine metabolism; pantothenate and CoA biosynthesis; beta-alanine, arginine, pyrimidine and proline metabolism; and steroid and aminoacyl-tRNA biosynthesis. In the NEG ion state, eleven possible pathways were discovered, as follows: cysteine, methionine, taurine, hypotaurine, glycine, serine, threonine, biotin, thiamine, phenylalanine, vitamin B6, and glutathione metabolism, and pantothenate, CoA, aminoacyl-tRNA, and primary bile acid biosynthesis.

## 4. Discussion

Previous studies in mouse models have demonstrated the beneficial effects of Pu-erh tea supplementation for the prevention of diet-induced obesity and related diseases [10,11]. However, studies on the effects of Pu-erh tea on host gut microbiota and metabolites and the possible underlying mechanisms are relatively scarce. The relationship between host gut microflora and metabolites has not been studied in depth. This study investigated the effects of CPC extract on body weight, GM, and faecal metabolites in HFD-supplemented mice. HFD induces obesity, insulin resistance, steatosis, and glucose intolerance. Regarding the results of CPC on body weight in HFD-supplemented mice, the CPC extract significantly reduced body and epididymal fat pad weight in HFD mice, and it attenuated daily energy expenditure in the HFD group, indicating that the effect of the CPC extract on body weight may be attributed to reduced energy intake. In addition, both the serum TC and LDL-C levels were reduced due to the CPC extract, but not in a statistically significant manner.

Obesity is caused by an excessive accumulation of fat in adipose tissue, which contains adipocytes and macrophages, which are closely associated with inflammation. Macrophages release inflammatory cytokines (TNF-α and IL-6), which are important immune effector cells [12]. Obesity can trigger local or systemic inflammation, and multiple inflammatory factors can cause chronic low-grade inflammation throughout the body [13]. Changes in serum concentrations of IL-6 and TNF-α indicate the presence of inflammation, and elevated serum levels of IL-6 and TNF-α are associated with chronic low-grade inflammation throughout the body. On the other hand, measuring hepatic levels of IL-6 and TNF-α allows us to understand the local production and activity of IL-6 and TNF-α directly in the liver. This may provide insights into the immune response of the liver and its involvement in various liver diseases such as hepatitis, cirrhosis, or liver cancer. This study showed that the CPC extract reduced serum IL-6 levels (*p* < 0.05). Although serum TNF-α levels and hepatic concentrations of TNF-α and IL-6 were lower in the CPC group than in the HFD group, they were not statistically significant.

The high-throughput sequencing data were analysed to determine the effect of the CPC extract on the composition and structure of the faecal microbiota. GM is critical for the progression of metabolic diseases [14]. HFD reproducibly induces obesity and metabolic syndrome in mice; the gut microbiome is a very important regulator of host metabolism, and its composition and functions are dynamic and influenced by dietary characteristics [15]. For example, dietary lipids are capable of altering host physiology by interacting with the GM [16]. The growth of the GM is influenced by lipids, which act as a substrate for metabolic pathways in bacteria and they inhibit bacterial growth. In mice and humans, GM affects lipid absorption and metabolism [17]. Moreover, disturbances in lipid metabolism in vivo also affect GM homeostasis [18].

In the results of CPC on GM in HFD-supplemented mice, supplementation with the CPC extract reversed the HFD-induced reduction in *Bacteroidetes* and increase in *Verrucomicrobia* at the phylum level in the gut of mice. In animal models, changes in the abundance of *Firmicutes* and *Bacteroidetes* are markers of metabolic disorders [19]. However, there is no clear evidence to suggest that altered *Firmicutes* and *Bacteroidetes* abundance ratios are associated with health status [20]. In the current study, the microbiota differed significantly at the genus level between the HFD and CPC groups, including *Akkermansia*, Alloprevotella, *Lactobacillus*, *Turicibacter*, *Bifidobacterium*, *Clostridium sensu stricto*, *Rikenella*, *Romboutsia*, *Allobaculum*, *Odoribacter,* and *Saccharibacteria_genera_incertae_sedis*, which were identified using LEfSe analysis. The association between the microbiota, which differed significantly at the genus level, and symptoms in obese individuals, was further investigated. In the human digestive tract, *Lactobacillus* spp. are among the most abundant microorganisms. However, the abundance of *Lactobacillus* spp. differs significantly between the intestines of healthy and diseased or compromised individuals, suggesting that some *Lactobacillus* spp. may be useful intestinal biomarkers [21]. The GM of animals that were fed a CPC extract is enriched with *Lactobacillus*. Moreover, the regulation of gut microbes by *Lactobacillus* provides anti-obesity benefits [22].

*Bifidobacterium* is a widespread genus belonging to the phylum *Actinomycetes*, found in the intestinal microbial flora of various animals, from insects to mammals, and it is thought to be beneficial to host health. Currently, *Lactobacillus* and *Bifidobacterium* are the most commonly used genera in probiotics. In addition, in this study, the abundance of *Bifidobacteria* in mice fed an HFD was significantly increased following treatment with the CPC extract. *Allobaculum*, a Gram-positive bacterium [23], can facilitate the interaction between GM and fat by inducing the expression of the angiopoietin-like protein 4, of which, butyric acid is a metabolite [24]. Angiopoietin-like protein 4 expression in colon cells is regulated by butyric acid through the transduction of PPARγ, which inhibits LPL expression, thereby exerting a regulatory effect on lipid metabolism [25]. In another study concerning the effects of berberine and metformin on obesity, *Allobaculum* was negatively associated with obesity and insulin resistance, as per the present research findings. Several studies have confirmed a significant reduction in *Turicibacter* abundance in the GM of HFD mice, with a notable negative association with body weight [26]. Since *Turicibacter* depolymerises most of the glycochenodeoxycholic acid and taurocholic acid when meals are consumed, it results in a high entry of BAs into the gut, and the levels of BAs in the gut regulate the abundance of *Turicibacter* [27].

*Turicibacter* is classified as an anti-inflammatory taxon [28]. The relative abundance of *Rikenella* was significantly lower in the GM of overweight or obese patients [29]. However, it has also been shown that the relative abundance of *Rikenella* was increased in HFD-supplemented male C57BL/6J mice [30]. Furthermore, in the present study, the CPC extract alleviated the HFD-induced reduction in *Rikenella* abundance. Thus, the CPC extract partially reversed the changes in the GM and weight gain induced by the HFD in mice in the current study.

Fecal metabolites are produced by the host and the GM, both of which are essential for maintaining health. In order to identify differential metabolites involved in the beneficial effects of the CPC extract, fecal samples were used to analyse variations in metabolic profiles and pathways. Regarding the results of CPC on fecal metabolites in HFD-supplemented mice, the cysteine and methionine pathway, which contains three differential metabolites (5′-methylthioadenosine, cysteic acid and L-cysteine), shows the most remarkable enrichment in the NEG ion mode. L-cysteine is a common amino acid in living organisms. However, with regard to this result, more differential amino acid metabolites were found, such as N-acetyl-L-tyrosine, L-threonine, and L-homocysteine isobutyrylglycine, N-acetylvaline, L-kynurenine, L-proline, D-proline, and so on. L-cysteine is an important substrate for maintaining intestinal function, and it is widely distributed in intestinal mucosal proteins and mucins. In this study, fecal L-cysteine levels were significantly increased in the CPC group after the intervention, in contrast to the HFD group. Some studies have shown that L-cysteine is essential for lipid metabolism, oxidative stress control, and growth promotion in vivo [31,32].

An important amino acid called threonine plays a role in protein synthesis, lipid metabolism, and intestinal health and function. Adequate levels of threonine help to alleviate energy metabolism disorders and intestinal inflammation [33]. The most significant enrichment in the POS ion pattern was observed in tryptophan metabolism, which includes four metabolites, as follows: melatonin, 3-hydroxyanthranilic acid, L-kynurenine, and formyl-5-hydroxykynurenamine. Accordingly, the CPC extract was found to have some effect on the amino acid metabolism in mice. As the most influential factor in the results of the differential pathway topology analysis, following treatment with CPC extract, the biotin pathway caused a remarkable reduction in fecal biotin content in HFD mice, with a possible increase in biotin utilisation in vivo. Biotin is a water-soluble vitamin, and most dietary biotin is absorbed in the small intestine, whereas biotin produced by gut bacteria is absorbed in the large intestine. A reduction in intestinal permeability, and the maintenance of mucosal integrity, was observed as biotin absorption and utilisation increased [34]. The analysis of metabolic pathway enrichment, primary bile acid, and steroid biosynthesis also underwent a noticeable change. Primary BAs are formed in the liver, whereas cholesterol is catalysed by the enzyme 7-α-hydroxylase to produce 7-α-hydroxycholesterol. Subsequently, primary BAs and chenodeoxycholic acid conjugate with glycine and taurine to form conjugated primary BAs [35]. In the liver, lipid homeostasis is affected by the primary bile acid metabolism, which, when abnormal, can lead to several diseases, including non-alcoholic fatty liver disease (NAFLD), primary amine oxidase (PrAO), cirrhosis, and obesity [36].

Caffeine metabolism in mice was also altered by treatment with CPC extract. In animals and humans, caffeine is mainly absorbed in the intestine and metabolised in the liver, from CYP1A2 to paraxanthine, theobromine, and theophylline [37]. In particular, *Pseudomonas putida*, CBB5, has been shown to biotransform caffeine and related natural purine alkaloids in the gut via the N-demethylase system [38]. Caffeine reduces HFD-induced lipid levels in mice, promotes lipolysis, and inhibits fat deposition; it also reduces intrahepatic lipid levels and it stimulates hepatocyte β-oxidation via the autolysosomal lysosomal pathway. In addition, caffeine promotes lipolysis by centrally stimulating catecholamine release and enhancing adipocyte responses to the adrenergic receptor-mediated activation of lipolysis. Caffeine also inhibits glucose transport in adipocytes, the primary step in adipogenesis [39]. Recent evidence has confirmed that caffeine inhibits insulin-stimulated basal glucose transport and adipogenesis in rodent adipocytes. In addition, caffeine inhibits the oxidation of phenylmethylamine via primary amine oxidase in human adipose tissue, thereby blocking the substrates of primary amine oxidase [40]. The reduction in body weight and adipose tissue in HFD-fed mice observed in this study may be attributed to the effect of the CPC extract on caffeine metabolism.

## 5. Conclusions

The effects of the CPC extract intervention on HFD mice include the following: (1) inhibition of abnormal weight gain and adipose tissue accumulation; (2) regulation of the relative abundance of certain functional GM, including *Lactobacillus*, *Bifidobacterium*, *Rikenella*, *Turicibacter* and *Allobaculum*; (3) effects on metabolic pathways in mice, including increases in theobromine levels regarding caffeine metabolism, increases in cysteic acid, l-cysteine, and 5′-methylthioadenosine, cysteine and methionine metabolism levels, increases in melatonin and formyl-5-hydroxykynurenamine levels, a reduction in 3-hydroxyanthranilic acid and l-kynurenine levels in tryptophan metabolism, a reduction in biotin levels in biotin metabolism, increased chenodeoxycholic acid levels in primary bile acid biosynthesis, and a reduction in 7-dehydrodesmosterol levels in steroid biosynthesis. The use of germ-free mice and faecal microbiota transplantation in subsequent studies will add to the available knowledge of the effects of the CPC extract on GM, and the effects of metabolite microbial interactions on the host’s metabolic health.

## Figures and Tables

**Table 1:** The composition of the high-fat diet and control diet.

Composition	Content (per 1000 g)	
	High-Fat Diet	Control Diet
Casein	233.06	189.58
Cystine	3.50	2.84
Corn starch	84.83	298.59
Maltose dextrin	116.53	33.18
Sucrose	201.36	331.77
Cellulose	58.26	47.40
Soybean oil	29.13	23.70
Lard	206.84	18.96
Mineral mixture M1002	11.65	9.48
Dicalcium phosphate	15.15	12.32
Calcium carbonate	6.41	5.21
Potassium citrate	19.23	15.64
Vitamin mixture V1001	11.56	9.48
Hydrocholine Tartaric acid	2.33	1.90
Edible red dye	0.058	0.047

## Data Availability

The 16S rRNA gene sequences were available at the NCBI Sequence Read Archive repository with Accession Code PRJNA817057. Untargeted metabolomic data have been deposited to the EMBL- EBI MetaboLights database with the identifier MTBLS4543, the complete data set can be accessed at www.ebi.ac.uk/metabolights/MTBLS4543 (accessed on 1 April 2022).

## Supplementary Materials

The following supporting information can be downloaded at: https://www.mdpi.com/article/10.3390/nu15163642/s1, Figure S1: PCA and OPLS-DA analysis of feces for the CPC versus HFD groups; Figure S2: Heatmap of the differential metabolites between the CPC extract and HFD groups in the POS ion mode; Figure S3: Heatmap of the differential metabolites between the CPC extract and HFD groups in the NEG ion mode.
