# Supplementary figures and images for: Modulatory Effects of Co-Fermented Pu-erh Tea with Aqueous Corn Silk Extract on Gut Microbes and Fecal Metabolites in Mice Fed High-Fat Diet
Source: Nutrients. 2023 Aug 19;15(16):3642. doi: 10.3390/nu15163642 (PMC10458734; doi:10.3390/nu15163642)

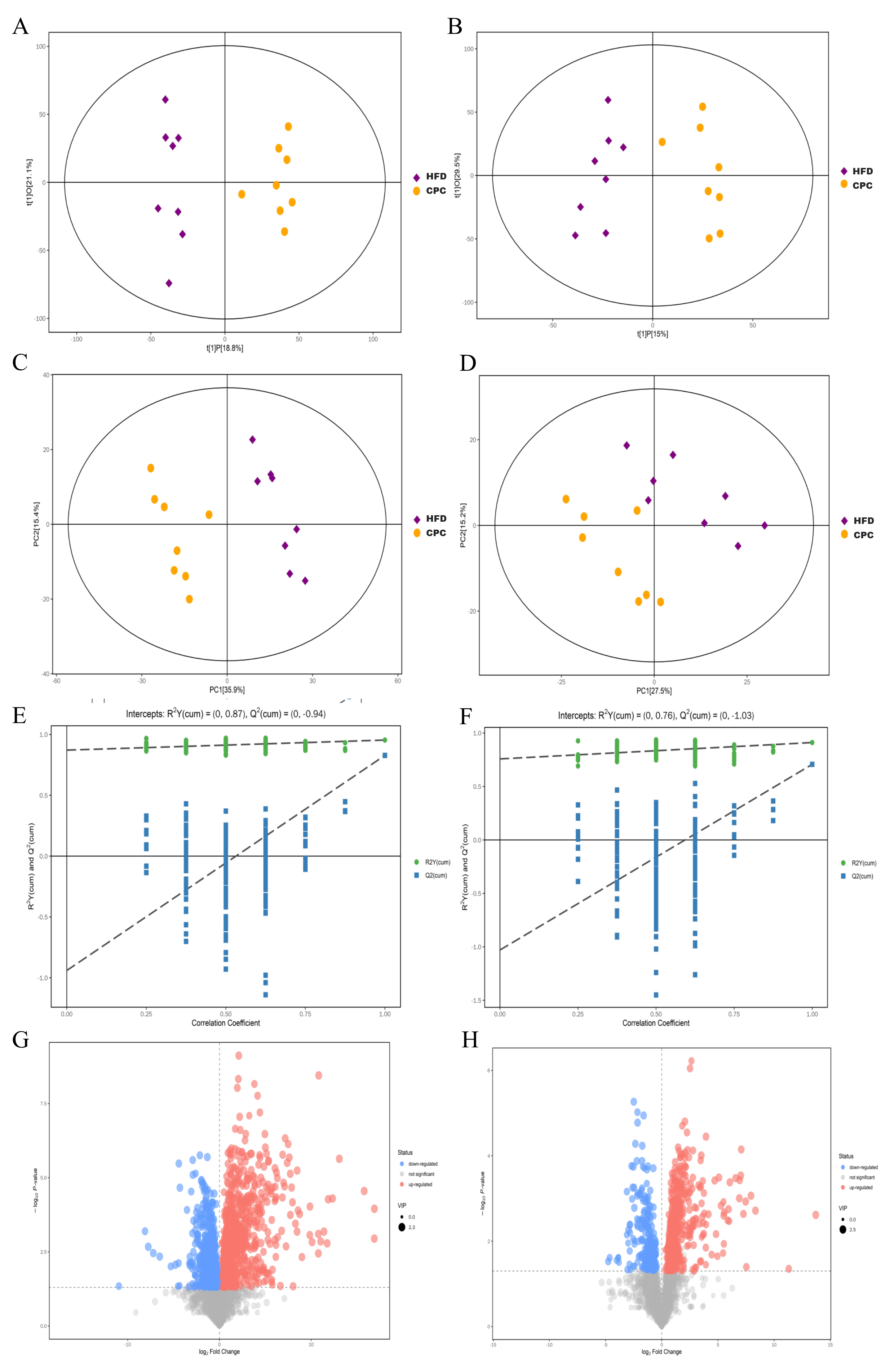

Supplement: Supplementary file 1 [file nutrients-15-03642-s001.zip › Supplementary Figure S1.tif]

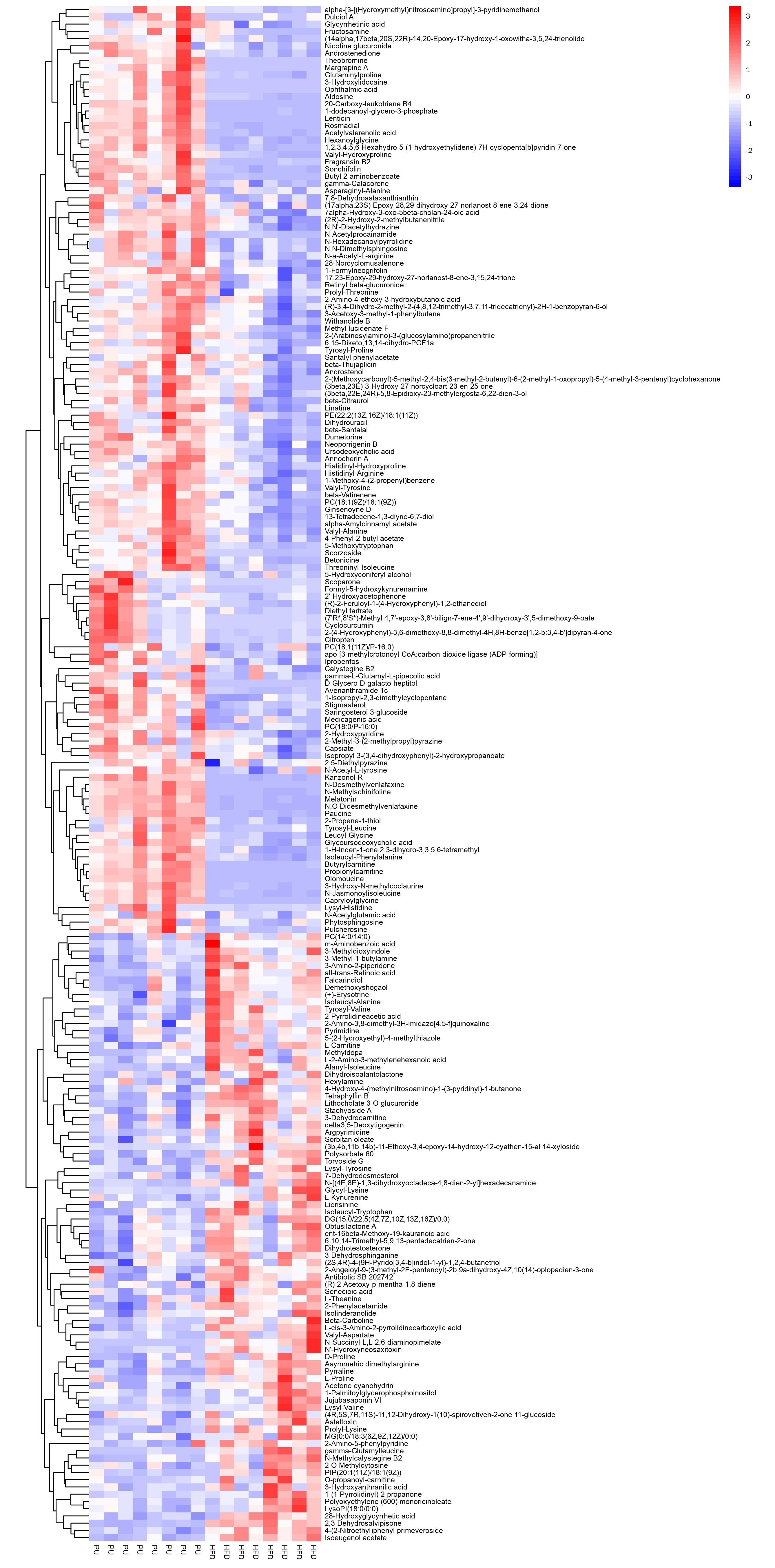

Supplement: Supplementary file 1 [file nutrients-15-03642-s001.zip › Supplementary Figure S2.jpg]

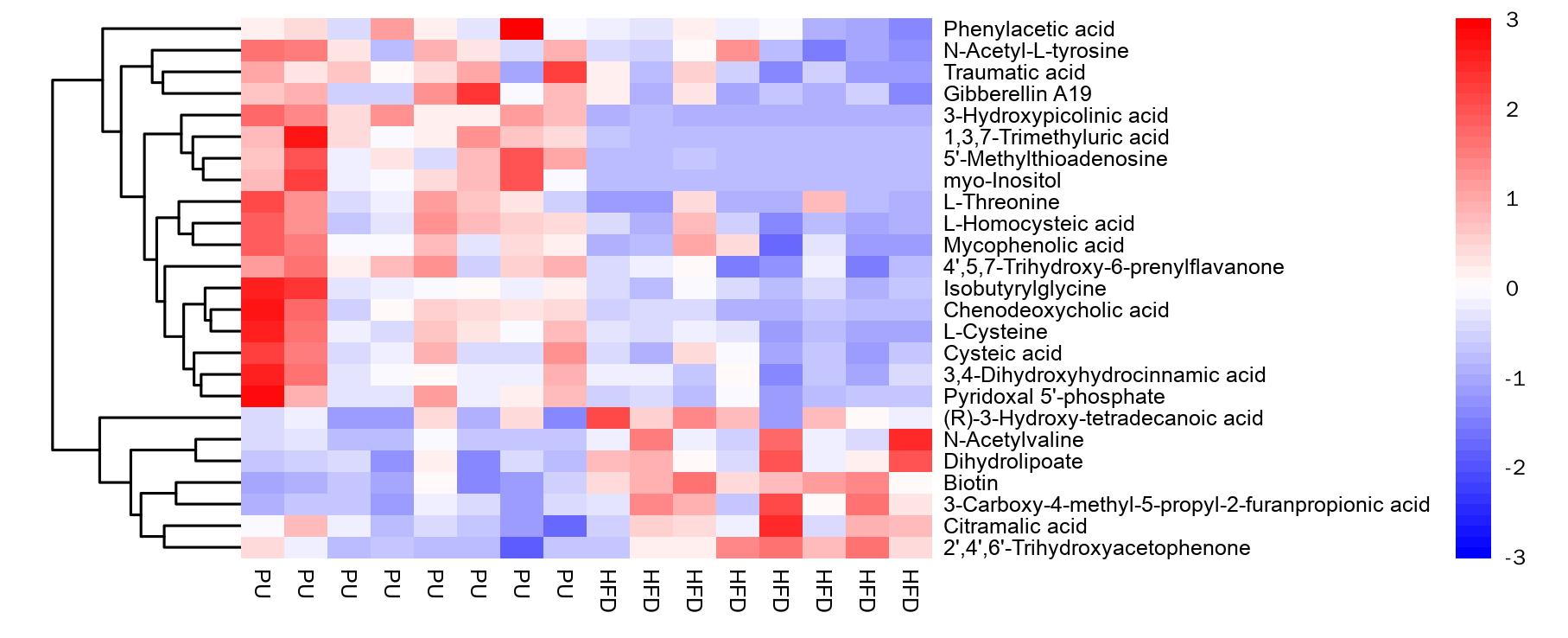

Supplement: Supplementary file 1 [file nutrients-15-03642-s001.zip › Supplementary Figure S3.jpg]
